# Supplementary material for: A set of multi-entry identification keys to African frugivorous flies (Diptera, Tephritidae)
Source: Zookeys. 2014 Jul 24;(428):97–108. doi: 10.3897/zookeys.428.7366 (PMC4143993; doi:10.3897/zookeys.428.7366)
Supplement: Supplementary material 6 — Key to Ceratitis [file zookeys-428-097-s006.zip › SF6_ZooKeys_key to Ceratitis/key/SF6_ZooKeys_key to Ceratitis/Media/Html/Ceratitis gravinotata .htm]

Ceratitis (Pterandrus) gravinotata (Munro)


***Ceratitis*** **(*Pterandrus*) *gravinotata*
(Munro)**

*Pterandrus gravinotatus* Munro, 1937: 9.

Body
length: 3.99 (3.25-4.45) mm; wing length: 4.12 (3.50-4.70) mm.

Male

Head: Antenna yellowish orange. First flagellomere three
times as long as pedicel. Arista with very short rays; basoventral rays shorter
and sparser than basodorsal rays. Frons on ventral half swollen; yellow,
lateral margin pale yellow; with short scattered setulae largely of same color
as frons. Frontal setae slightly less developed than other setae. Face
yellowish white; gena with yellowish brown markings. Genal seta and setulae
dark, well developed.

Thorax:
Postpronotal lobe yellowish white, with dark spot. Mesonotum: ground color
brown, microtrichose areas silvery with ashgray shine, spots brownish
black, prescutellar yellow markings separate. Scapular setae dark. Scutellum
yellowish white, basally with two separate dark spots, apically with three
merged black spots, extending to basal spots and touching, incised to basal
half, sometimes less. Anepisternum on ventral 0.33 brown; setulae pale except
on ventral 0.33 where black.

Legs:
Yellowish orange except where otherwise noted; setation typical for subgenus,
dark and pale. Foreleg: femur brown, posteriorly with dispersed bush of long
dark setulae along entire length, posterodorsal setulae longer; ventral setae
dark. Midleg: femur darker yellow, sometimes orange-brown; basally few short
dark setulae, not always conspicuous; ventrally with dispersed dark setulae and
setae; tibia orange-brown and dilated on ventral 0.66; ventrally and dorsally
with dark feathering along distal 0.66; indication of third row anterior of
dorsal feathering but not developed. Hindleg: femur partly orange-brown; at
apical 0.25 with longer setulae dorsally and ventrally.

Wing:
bands brownish. Interruption between marginal and discal bands narrow, not
always complete; cubital band free; medial band joined with marginal,
occasionally free; crossvein R-M opposite middle of discal cell. Apex of vein R_1_
distal to level of crossvein R-M. Crossvein DM-Cu oblique anterobasally.

Abdomen:
Mostly brown. Tergite 1 yellow along anterior half. Tergites 2 across posterior
half to 0.66 and tergite 4 wholly with silvery band. Tergite 5 sometimes with
paler patches. Male epandrium in lateral view with lateral surstylus slender
and elongate, strongly curved.

Female

As
male except for the following characters: First flagellomere twice or three
times as long as pedicel, darker color. Frontal setae well developed. Legs
without feathering; orange-brown except tarsi and foretibia yellow; forefemur
posteroventrally with dark pilosity. Wing with narrow but complete interruption
marginal and discal bands; medial band sometimes weakly developed. Tergite 5
across posterior half with silvery band. Oviscape shorter than preabdomen.
Aculeus about six times longer than wide; tip pointed and lateral margin
concave.

(Description
after De Meyer & Freidberg, 2006)
